# Enterobius vermicularis in Paediatric Appendicectomy Patients in Far North Queensland, Australia: A Retrospective Study

**DOI:** 10.7759/cureus.103743

**Published:** 2026-02-16

**Authors:** Sophia Chan, Elena Pilat, Juyong Cheong, Christian Beardsley

**Affiliations:** 1 General Surgery, Cairns Hospital, Cairns, AUS

**Keywords:** acute appendicitis diagnosis, emergency appendicectomy, enterobius vermicularis infestation, paediatric appendicitis, rural surgery

## Abstract

Background

*Enterobius vermicularis *(*E. vermicularis*) is a common parasitic infection in children and is frequently identified incidentally in appendicectomy specimens. Its role in the pathogenesis of acute appendicitis remains controversial, with many cases demonstrating minimal or no appendiceal inflammation. Australian data describing the prevalence and clinical significance of *E. vermicularis* in paediatric appendicitis are limited.

Methods

A retrospective study was conducted of all patients aged <16 years who underwent appendicectomy at Cairns Hospital, a regional tertiary centre in Far North Queensland (FNQ), between January 2020 and December 2024. Demographic data, laboratory markers (white cell count {WCC} and C-reactive protein {CRP}), intraoperative findings and histopathology were extracted from electronic medical records. Intraoperative severity was graded using a four-level classification. Statistical analyses compared clinical and pathological features of *E. vermicularis*-positive cases.

Results

A total of 455 paediatric appendicectomies were performed during the study period. Of these patients, 370 cases (81%) had a positive histopathology for acute appendicitis. *Enterobius vermicularis* was identified in 45 specimens (9.9%). Among *E. vermicularis*-positive cases, 17 cases (37.8%) had a macroscopically normal appendix intraoperatively, and uncomplicated appendicitis was the most common operative impression (25 cases, 55.6%). Histopathology demonstrated acute inflammatory appendicitis in 19 out of 45 cases (42%) of *E. vermicularis*-positive specimens, while the remainder of the specimens were completely normal. Leucocytosis was present in 17 (37.8%) out of 45 cases, and C-reactive protein (CRP) was elevated in 26 (60.4%) cases, with CRP results unavailable for two patients.

Conclusion

*Enterobius vermicularis* was identified in approximately one in 10 paediatric appendicectomy specimens in Far North Queensland, a prevalence higher than that reported in most contemporary cohorts. The majority of *E. vermicularis*-positive cases lacked histological evidence of acute appendicitis, supporting the concept of appendiceal colic mimicking appendicitis. The routine postoperative review of histopathology is essential to ensure appropriate antiparasitic treatment and household management. The increased clinical awareness of parasitic mimics may help inform diagnostic decision-making and, if accompanied by appropriate blood tests and imaging, can reduce unnecessary surgery in selected low-risk children.

## Introduction

Acute appendicitis remains the most common indication for emergency abdominal surgery in children and is frequently diagnosed on the basis of clinical features supported by laboratory and imaging findings. Clinical features generally include periumbilical pain that is migratory to the right iliac fossa, nausea or vomiting, loose stools and/or fevers. However, right iliac fossa pain has a broad differential diagnosis, and negative appendicectomy (histologically normal appendix) remains an important quality and safety issue [1].

*Enterobius vermicularis *(*E. vermicularis*) (threadworm/pinworm) is a ubiquitous nematode that predominantly affects preschool and school-aged children and spreads readily within households and crowded settings. A recent systematic review estimated a pooled global prevalence of enterobiasis in children of approximately 4%-28%, although individual studies vary widely by geography and diagnostic method [2]. Infection occurs via the ingestion or inhalation of embryonated eggs, with autoinfection facilitating persistent household transmission; eggs can survive in the environment for up to two weeks.

Most infections are asymptomatic, but classic symptoms include nocturnal perianal pruritus, sleep disturbance, irritability and, in girls, vulvovaginitis [3,4]. Diagnosis is commonly made using the perianal ‘tape test’ because stool specimens often lack sufficient eggs for microscopy [3]. Standard therapy involves an anthelmintic dose repeated after two weeks, in conjunction with the treatment of household contacts and environmental hygiene measures to minimise reinfection [3,4].

Since the first descriptions of pinworm within appendicectomy specimens in the late 19th century, the relationship between *E. vermicularis* and acute appendicitis has been debated [5-7]. Proposed mechanisms include luminal obstruction, local mucosal irritation or egg deposition with secondary bacterial infection; however, many resected appendices containing *E. vermicularis* show either no acute inflammation or only chronic/eosinophil-predominant changes, supporting the concept of ‘appendiceal colic’ that clinically mimics appendicitis [6,7].

A global systematic review and meta-analysis including over 100,000 appendix tissue samples reported a pooled prevalence of *E. vermicularis* of 4,000 (4%) among appendicitis cases, with considerable heterogeneity between regions and a higher prevalence in lower-income settings [5]. Contemporary cohort studies in high-income settings generally report *E. vermicularis *in <1%-7% of appendicectomy specimens and consistently show a high proportion (up to 69%) of histologically normal appendices in *E. vermicularis*-positive patients [8-10].

Australian data describing the frequency of *E. vermicularis* in appendicectomy specimens are scarce. Far North Queensland (FNQ) is a distinct setting due to its tropical climate, large geographic catchment and substantial Aboriginal and Torres Strait Islander paediatric population, in whom broader enteric and parasitic infections remain important health issues in some remote communities [11,12]. Local epidemiology may therefore influence the likelihood of *E. vermicularis* presenting as a mimic of appendicitis or coexisting with true acute appendicitis.

The primary aim of this study was to determine the prevalence of *E. vermicularis* in paediatric appendicectomy specimens at a single regional tertiary centre (Cairns Hospital) between January 2020 and December 2024. Secondary aims were to describe the associated inflammatory markers, intraoperative grading and histopathological diagnosis of appendicitis; inform postoperative follow-up; and identify potential opportunities to reduce unnecessary surgery.

## Materials and methods

Study setting, design and exclusion criteria

This retrospective, single, tertiary regional institution study included all patients aged <16 years who underwent appendicectomy at Cairns Hospital between January 2020 and December 2024. Eligible cases were identified through procedure coding in the electronic medical record. Exclusion criteria included patients managed non-operatively with antibiotics and those who underwent interval appendicectomy. The patients who were managed non-operatively were not included, as there is no formal coding system that can identify these patients.

Data collection

Electronic medical records for each patient were reviewed. Data extracted included patient demographics (age, sex and Aboriginal and Torres Strait Islander status), laboratory markers (white cell count {WCC}, reference range: 4.5-13.5 × 10^9^/L; C-reactive protein {CRP}, reference range: 0-3.0mg/L), intraoperative findings recorded using a four-level grading system and histopathology (normal appendix, acute appendicitis and the presence of *E. vermicularis*) [13]. Intraoperative severity was graded as grade 1, macroscopically normal appendix; grade 2, uncomplicated appendicitis without contamination; grade 3, appendicitis with localised peritonitis; and grade 4, appendicitis with generalised peritonitis. This particular grading system is based on a multicentre observational study published in the World Journal of Emergency Surgery [14].

Statistical analysis

Continuous variables were summarised using mean (standard deviation {SD}) or median (interquartile range {IQR}) depending on distribution and compared using t-tests or Mann-Whitney U tests. Categorical variables were compared using χ^2^ tests or Fisher’s exact tests.

Ethics statement

This study was approved by the Far North Queensland Human Research Ethics Committee (FNQ HREC) (approval number: EX/2025/QCH/119144). This retrospective study only contains existing, de-identified data; therefore, a waiver of consent is permitted.

## Results

Patient characteristics

Over the five-year study period, 455 paediatric patients underwent an appendicectomy at the Cairns Hospital. The mean age was 11.0 years (SD: 3.0), and 269 (59.1%) were men. Aboriginal and Torres Strait Islander patients comprised 137 cases (30.1%). The number of cases positive for *E. vermicularis* was identified in 45 cases (9.9%).

Laboratory markers

Among *E. vermicularis*-positive cases, leucocytosis was present in 17 (37.8%) out of 45 cases. C-reactive protein (CRP) was elevated in 26 (60.4%) cases, with CRP results unavailable for two patients.

Intraoperative findings

Among *E. vermicularis*-positive cases, 17 cases (37.8%) had a macroscopically normal appendix (grade 1). The most common intraoperative impression was uncomplicated appendicitis without contamination (grade 2) in 25 cases (55.6%), while localised peritonitis/contained perforation (grade 3) and generalised peritonitis (grade 4) were uncommon, with only two cases (4.4%) and one case (2.2%), respectively. The proportion of positive appendicitis specimens was 23.53% for grade 1, 48% for grade 2 and 100% for grade 3 and 4 cases. Please refer to Table 1, which shows intraoperative findings, the number of cases and corresponding appendicitis on histopathology, for a structured representation of the aforementioned results.

**Table 1:** Intraoperative findings, number of cases and corresponding appendicitis on histopathology (%)

Intraoperative findings	Number of cases (%)	Histopathology (percent of appendicitis cases)
Grade 1: macroscopically normal appendix	17/45 (37.78%)	4/17 (23.53%)
Grade 2: appendicitis with no contamination	25/45 (55.56%)	12/25 (48%)
Grade 3: appendicitis with localised/contained perforation	2/45 (4.4%)	2/2 (100%)
Grade 4: appendicitis with gross peritonitis	1/45 (2.2%)	1/1 (100%)

Histopathology

There were 370 cases (81%), which had a positive histopathology for acute appendicitis and, therefore, a negative appendicectomy rate of 85 cases (19%). Of the *E. vermicularis*-positive cases, 19 cases (42%) showed acute inflammatory appendicitis; the remainder had no histological evidence of acute appendicitis.

## Discussion

This retrospective series demonstrates that *E. vermicularis* is a relatively frequent histopathological finding in paediatric appendicectomy specimens in FNQ, identified in around 45 (10%) operations for suspected appendicitis. Importantly, fewer than half of *E. vermicularis*-positive specimens demonstrated histological acute appendicitis, and a substantial proportion was macroscopically and histologically normal.

The observed prevalence is higher than that reported in most contemporary paediatric appendicectomy cohorts, where rates typically range from 0.6% to 1.1% and rarely exceed 7% [8-10]. For example, Pogorelić et al. [8] identified *E. vermicularis* in 0.96% of 6,359 paediatric appendectomies, while Sousa et al. [9] reported 1.07% in a 22-year series; Fleming et al. [10] reported a higher rate of 7% in an Irish paediatric cohort. At a global level, Taghipour et al. estimated a pooled prevalence of 4% among appendicitis cases, with substantial geographic heterogeneity [5]. Potential explanations for the higher proportion in FNQ include differing baseline community prevalence, household crowding and hygiene factors, differences in the age distribution of surgical patients and local pathology practices that may increase detection.

Our finding that most *E. vermicularis*-positive appendices were normal or only mildly inflamed aligns with earlier histopathological series, which concluded that *E. vermicularis* is rarely associated with acute appendicitis and more commonly linked to appendicular pain without inflammation [6-10]. This supports the concept of appendiceal colic, in which luminal irritation or transient obstruction produces symptoms that are clinically indistinguishable from appendicitis, leading to potentially avoidable surgery [7].

Generally, there are two intraoperative approaches to a macroscopically normal-appearing appendix; proceeding with an appendicectomy excludes a diagnostic dilemma in the future; however, one can also justify not removing an appendix (or other organs) that appears normal. Acknowledging a 10% prevalence (10 times higher than the reported prevalence in other international cohorts) of *E. vermicularis*-positive cases in FNQ, the removal of macroscopically normal appendices may be justified.

Nevertheless, 42% of *E. vermicularis*-positive specimens in our cohort showed histologically acute appendicitis. Similar proportions have been reported in paediatric cohorts that compared infestation without appendicitis to *E. vermicularis*-associated appendicitis, suggesting that the parasite may coexist with, precipitate or simply be an incidental bystander in bacterial appendicitis [8-10]. Proposed mechanisms include luminal obstruction by worms or eggs, mucosal injury and secondary bacterial invasion; however, retrospective histopathology alone cannot establish causality [5-7].

Inflammatory markers were frequently normal in our *E. vermicularis*-positive patients (WCC normal in 62% and CRP normal in 40%), consistent with the observation that patients with infestation alone often have lower systemic inflammatory responses than those with true appendicitis [8,10]. Pogorelić et al. reported significantly lower WCC, neutrophils and CRP and lower Appendicitis Inflammatory Response (AIR) scores in children with infestation without appendicitis compared to those with *E. vermicularis*-associated acute appendicitis [8]. The routine documentation of eosinophil counts and specific symptoms (e.g. nocturnal pruritus and household contacts with pinworm) may further improve preoperative suspicion, although eosinophilia is inconsistently present in published series [3,8].

Most *E. vermicularis*-positive cases were graded intraoperatively as either normal (grade 1) or uncomplicated appendicitis without contamination (grade 2), with perforated disease being uncommon. This pattern is in keeping with other series where infestation is often discovered in macroscopically normal appendices and may reflect a lower likelihood of severe inflammatory disease when *E. vermicularis* is the primary driver of symptoms [8-10].

The principal clinical implication of our findings is the importance of the postoperative review of histopathology. Identifying *E. vermicularis* provides an opportunity to treat the patient and household contacts with an anthelmintic regimen (with a second dose at two weeks) and implement hygiene measures to reduce reinfection and ongoing transmission [3,4]. In practice, this may require a clear communication pathway between surgical teams, primary care provider and families, particularly for patients from remote communities.

In settings where children present with right iliac fossa pain but have low inflammatory markers, a benign abdominal examination and imaging that does not support appendicitis, *E. vermicularis* should be considered as part of the differential diagnosis [3,8]. While appendicectomy remains appropriate when clinical concern for appendicitis is high, the heightened awareness of parasitic mimics may support a period of observation and repeat assessment in selected low-risk patients, potentially reducing negative appendicectomy [1,7].

Although direct Australian prevalence data for enterobiasis are limited, enteric and parasitic infections continue to be reported in some remote Aboriginal and Torres Strait Islander communities, reflecting complex interactions between environmental exposure, housing and sanitation [11,12]. Understanding local epidemiology may help clinicians in FNQ calibrate pretest probabilities for alternative diagnoses and reinforces the value of culturally appropriate community health interventions alongside individual treatment.

This study has several limitations. Its retrospective, single-centre design limits causal inference and generalisability. Case identification relied on administrative procedure coding, and we did not include children managed non-operatively, potentially introducing selection bias. Data on specific symptoms of enterobiasis (perianal pruritus and sleep disturbance) and eosinophil counts were incomplete, and there was no matched *E. vermicularis*-negative comparator group to quantify differences in laboratory or clinical presentation. Finally, the presence of *E. vermicularis *on histopathology may represent incidental carriage rather than aetiological relevance to the presenting episode.

Prospective studies that systematically capture symptom profiles, eosinophil counts, imaging findings and microbiology and that include non-operative management pathways are needed to better define when *E. vermicularis* is a true contributor to appendicitis versus a mimic. Such work could inform decision support tools and postoperative follow-up processes in regions where enterobiasis is more prevalent.

## Conclusions

*Enterobius vermicularis* is a clinically relevant finding in paediatric appendicectomy patients in Far North Queensland. Many *E. vermicularis*-positive cases demonstrated no macroscopic or histological appendicitis, suggesting that the parasite may mimic appendicitis rather than cause true inflammation in a subset. Increased clinical awareness and routine follow-up of histopathology may help identify children who would benefit from antiparasitic therapy. Further research involving multiple centres and the comparison of cohorts who underwent non-operative management can provide valuable insight into this controversial topic.
